# Cardiac (myo)fibroblasts modulate the migration of monocyte subsets

**DOI:** 10.1038/s41598-018-23881-7

**Published:** 2018-04-03

**Authors:** Kathleen Pappritz, Konstantinos Savvatis, Annika Koschel, Kapka Miteva, Carsten Tschöpe, Sophie Van Linthout

**Affiliations:** 1 Charité – Universitätsmedizin Berlin, corporate member of Freie Universität Berlin, Humboldt-Universität zu Berlin, and Berlin Institute of Health, Department of Internal Medicine and Cardiology, Campus Virchow Klinikum, Berlin, Germany; 2 DZHK (German Center for Cardiovascular Research), partner site Berlin, Berlin, Germany; 3 Charité – Universitätsmedizin Berlin, corporate member of Freie Universität Berlin, Humboldt-Universität zu Berlin, and Berlin Institute of Health, Berlin-Brandenburg Center for Regenerative Therapies, Campus Virchow Klinikum, Berlin, Germany; 4 Inherited Cardiovascular Diseases Unit, Barts Health NHS Trust, Barts Heart Centre, London, UK; 5 William Harvey Research Institute, Queen Mary University London, London, UK; 6 Department of Biomedical Sciences, Humanitas University; Adaptive Immunity Laboratory, Humanitas Clinical and Research Center Pieve Building, Rozzano, Milano, Italy

## Abstract

Cardiac fibroblasts play an important role in the regulation of the extracellular matrix and are newly recognized as inflammatory supporter cells. Interferon (IFN)-γ is known to counteract transforming growth factor (TGF)-ß1-induced myofibroblast differentiation. This study aims at investigating *in vitro* how IFN-γ affects TGF-ß1-induced monocyte attraction. Therefore, C4 fibroblasts and fibroblasts obtained by outgrowth culture from the left ventricle (LV) of male C57BL6/j mice were stimulated with TGF-β1, IFN-γ and TGF-β1 + IFN-γ. Confirming previous studies, IFN-γ decreased the TGF-ß1-induced myofibroblast differentiation, as obviated by lower collagen I, III, α-smooth muscle actin (α-SMA), lysyl oxidase (Lox)-1 and lysyl oxidase-like (LoxL)-2 levels in TGF-β1 + IFN-γ- versus TGF-ß1-stimulated cardiac fibroblasts. TGF-β1 + IFN-γ-stimulated C4 and cardiac fibroblasts displayed a higher CC-chemokine ligand (CCL) 2, CCL7 and chemokine C-X3-C motif ligand (Cx3CL1) release versus sole TGF-ß1-stimulated fibroblasts. Analysis of migrated monocyte subsets towards the different conditioned media further revealed that sole TGF-β1- and IFN-γ-conditioned media particularly attracted Ly6C^low^ and Ly6C^high^ monocytes, respectively, as compared to control media. In line with theses findings, TGF-β1 + IFN-γ-conditioned media led to a lower Ly6C^low^/Ly6C^high^ monocyte migration ratio compared to sole TGF-ß1 treatment. These differences in monocyte migration reflect the complex interplay of pro-inflammatory cytokines and pro-fibrotic factors in cardiac remodelling and inflammation.

## Introduction

Cardiac fibroblasts are centrally involved in myocardial remodelling^1^. While their main role is traditionally believed to be the regulation of the extracellular matrix proteins, their function as inflammatory supporter cells has attracted recognition^2,3^. During the development of heart failure, the immune system is activated locally in the myocardium as well as systemically. The following interaction of the different cellular and humoral components of the immune system with the cardiac innate cells orchestrates the myocardial remodelling and compensatory mechanisms^4^. There is currently a growing body of evidence that the immune system exhibits significant effects on cardiac fibroblasts by controlling their activation and transformation into myofibroblasts^3,5^.

TGF-β1, secreted by immune cells, cardiac fibroblasts and cardiomyocytes^6^, induces myofibroblast transdifferentiation and matrix deposition, characterized by the enhancement of collagen I, collagen III and α-SMA, and it is involved in cell proliferation, endothelial-to-mesenchymal transition^7^, immune suppression and inflammation^8^. Maintenance of the extracellular matrix and myofibroblast persistence is essential to preserve functional cardiac integrity after ischemic injury^9^. IFN-γ is the key cytokine in the Th1 reaction and is produced in large amounts by different cell types, mainly CD4^+^ helper T cells, CD8^+^ cytotoxic T cells and natural killer (NK) cells^10^. In a previous study, we demonstrated that increased levels of IFN-γ and an excessive Th1 reaction after myocardial infarction leads to decreased myofibroblast differentiation with detrimental effects in scar formation and myocardial remodelling^11^. This supports the general consensus that an extended or prolonged inflammation results in inadequate myocardial wound healing and scar formation associated with a suppression of systolic function and chamber dilation^12^.

Besides T and NK cells, monocytes also play a pivotal role in cardiac remodelling during viral-induced inflammation^13^ and myocardial infarction^14^. In general, they can be divided into two main subsets identified by the expression of Ly6C. Pro-inflammatory monocytes are Ly6C^high^ expressing cells, whereas their anti-inflammatory and reparative counterparts are characterized by Ly6C^low^ expression. The chemokines CCL2 and CCL7 mediate Ly6C^high^ monocyte recruitment, whereas Cx3CL1 governs Ly6C^low^ infiltration into the myocardium^15^. In Coxsackievirus B (CVB) 3-induced myocarditis, Müller and co-authors could demonstrate that ablation of CX3CR1 resulted in an increased level of CCL2 accompanied by higher numbers of monocytes/macrophages^16^, further indicating the relevance of the chemokines CCL2 and Cx3CL1 in the attraction of monocytes.

Based on the finding that IFN-γ counteracts TGF-ß1-induced cardiac fibrosis^11^ on the one hand, and the relevance of monocyte subpopulations during inflammation and wound healing on the other hand^15^, we sought to examine *in vitro* how IFN-γ can influence the chemokine profile of cardiac (myo)fibroblasts and subsequent attraction of pro-inflammatory versus anti-inflammatory monocytes.

## Results

### IFN-γ reduces TGF-ß1-induced collagen production and myofibroblast differentiation

**Figure 1:**
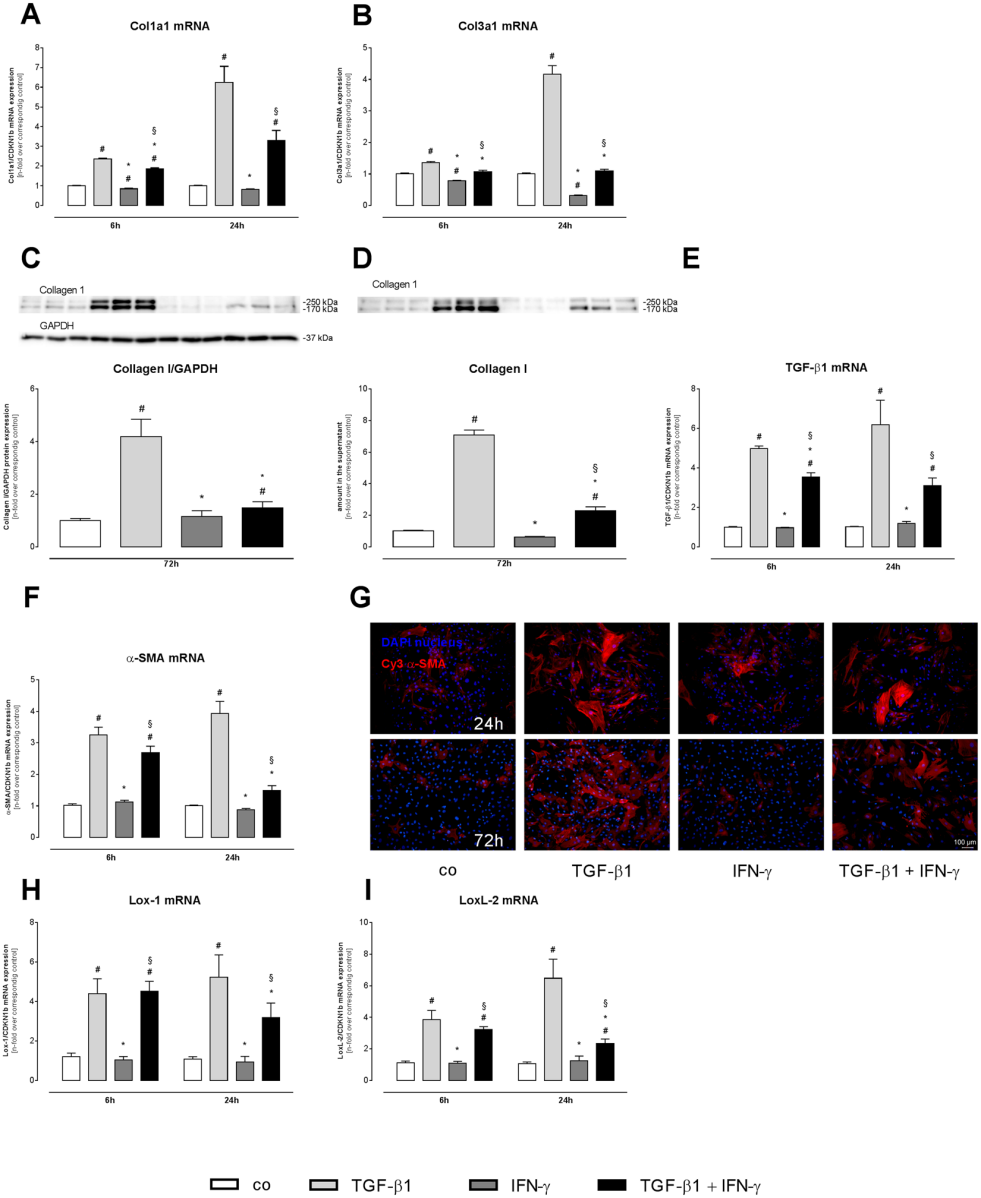
IFN-γ reduces TGF-ß1-induced transdifferentiation of cardiac fibroblasts. Data show the gene expression of the extracellular matrix proteins Col1a1 (**A**) and Col3a1 (**B**) and myofibroblast markers TGF-ß1 (**E**) and α-SMA (**F**) after 6 and 24 h incubation with or without TGF-ß1 and/or IFN-γ. Additionally, representative western blots (**C**,**D**) and quantification of Collagen I in cell lysate (**C**) or supernatant (**D**) after 72 h stimulation are depicted. (**G**) α-SMA marked by a Cy3-conjugated streptavidin antibody (red) and DAPI for nuclear staining (blue). Recording of the images was done in a 100× magnification (scale bar = 100 μm). To characterize collagen crosslinking, the gene expression of the crosslinking enzymes (**H**) Lox-1 and (**I**) LoxL-2 were measured. Bar graphs represent the mean ± SEM of 3 independent experiments after normalization to the corresponding control fibroblasts. Statistical analysis was performed by One way-ANOVA or Kruskal Wallis test (^#^p < 0.05 vs co, *p < 0.05 vs TGF-β1, ^§^p < 0.05 vs IFN-γ).

Before evaluating the impact of IFN-γ on the TGF-β1-mediated chemokine response and subsequent effect on the attraction of monocyte subsets, the impact of TGF-β1 + IFN-γ on collagen production and myofibroblast differentiation was determined. Confirming previous findings^17^, 24 h stimulation of cardiac fibroblasts with TGF-β1 led to a 6.2-fold (p < 0.0001), 4.1-fold (p < 0.0001), 3.9-fold (p < 0.0001) and 6.1-fold (p < 0.0001) higher collagen 1a1, collagen 3a1, TGF-ß1, and α-SMA mRNA expression as compared respectively to control cardiac fibroblasts, which is indicative for myofibroblast differentiation (Fig. 1A,B,E,F). Additional treatment with IFN-γ abrogated the TGF-ß1-mediated increase, leading to 3.8-fold (p < 0.001) and 2.6-fold (p < 0.001) lower collagen 3a1 and α-SMA mRNA expression levels 24 h post stimulation as compared respectively to sole TGF-β1-stimulated cardiac fibroblasts (Fig. 1B,F). On protein level, a 4.2-fold (p < 0.0001) increased expression of collagen I was observed in TGF-β1-treated cardiac fibroblasts versus control cardiac fibroblasts (Fig. 1C). In parallel, the amount of secreted collagen I in the cell culture supernatant was increased by 7.1-fold (p < 0.0001) after sole TGF-β1 stimulation in comparison to control cardiac fibroblasts (Fig. 1D). Further evaluation of the collagen crosslinking enzymes Lox-1 and LoxL-2 demonstrated that TGF-ß1 induced Lox-1 and LoxL-2 gene expression versus control cardiac fibroblasts (Lox-1: 4.8-fold, p < 0.0001; LoxL-2: 6.1-fold, p < 0.0001) 24 h post stimulation (Fig. 1H,I).

Compared to sole TGF-ß1 stimulation, administration of TGF-ß1 + IFN-γ resulted in a 2.8-fold (p < 0.0001) decreased collagen I protein expression in cardiac fibroblasts and a 3.1-fold (p < 0.0001) reduced collagen I amount in cell culture supernatant (Fig. 1C,D). Immunofluorescence staining further showed a significant reduction of α-SMA protein expression after treatment with both stimuli compared to single TGF-ß1 treatment (Fig. 1G). With respect to Lox-1 and LoxL-2 expression, 24 h co-stimulation with TGF-ß1 + IFN-γ led to 1.6-fold (p < 0.05) and 2.8-fold (p < 0.05) lower Lox-1 and LoxL-2 mRNA levels as compared respectively to sole TGF-ß1 administration (Fig. 1H,I).

### IFN-γ induces an inflammatory phenotype in cardiac fibroblasts

**Figure 2:**
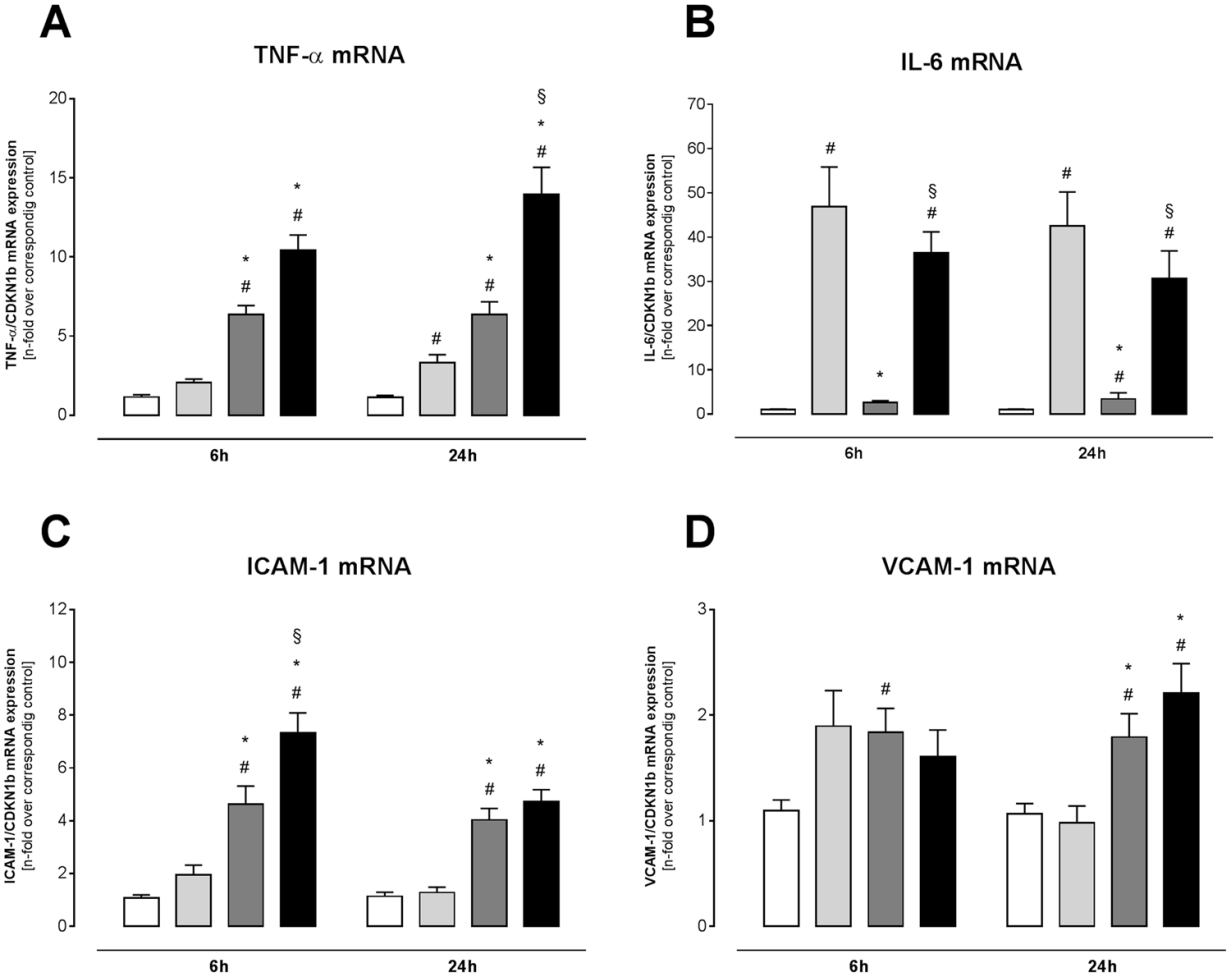
IFN-γ modulates TGF-ß1-induced cytokine and adhesion molecule expression of cardiac fibroblasts. To determine the inflammatory phenotype after stimulation with TGF-ß1, IFN-γ, or both, the mRNA expression of (**A**) TNF-α, (**B**) IL-6, (**C**) ICAM-1, and (**D**) VCAM-1 was investigated. Bar graphs represent the mean ± SEM of 3 independent experiments after normalization to the corresponding control fibroblasts. Statistical analysis was performed by One way-ANOVA or Kruskal Wallis test (^#^p < 0.05 vs co, *p < 0.05 vs TGF-β1, ^§^p < 0.05 vs IFN-γ).

Before assessing the impact of TGF-ß1, IFN-γ, and TGF-ß1 + IFN-γ stimulation on cardiac fibroblast chemokine expression, we evaluated how these stimuli could affect the cardiac fibroblasts as inflammatory supporter cells in terms of cytokine and adhesion molecule expression (Fig. 2). Therefore, gene expression of tumor necrosis factor (TNF)-α, interleukin (IL)-6, intercellular adhesion molecule (ICAM)-1 and vascular adhesion molecule (VCAM)-1 was measured. TGF-ß1 treatment alone led to a 3.0-fold (p < 0.05) and 39.6-fold (p < 0.0001) increase in TNF-α and IL-6 mRNA expression versus control fibroblasts after 24 h (Fig. 2A,B), respectively, whereas no significant change in the expression of ICAM-1 and VCAM-1 was detected (Fig. 2C,D). Interestingly, simultaneous treatment with TGF-ß1 + IFN-γ caused a 3.7-fold (p < 0.0001) and 2.3-fold (p < 0.001) induction of ICAM-1 and VCAM-1 mRNA in comparison to TGF-β1 alone (Fig. 2C,D). Furthermore, TGF-ß1 + IFN-γ boosted the increased expression of TNF-α, as indicated by a 4.2-fold (p < 0.0001) elevation compared to TGF-β1 administration.

### IFN-γ further modulates chemokine expression in TGF-ß1 stimulated fibroblasts

**Figure 3:**
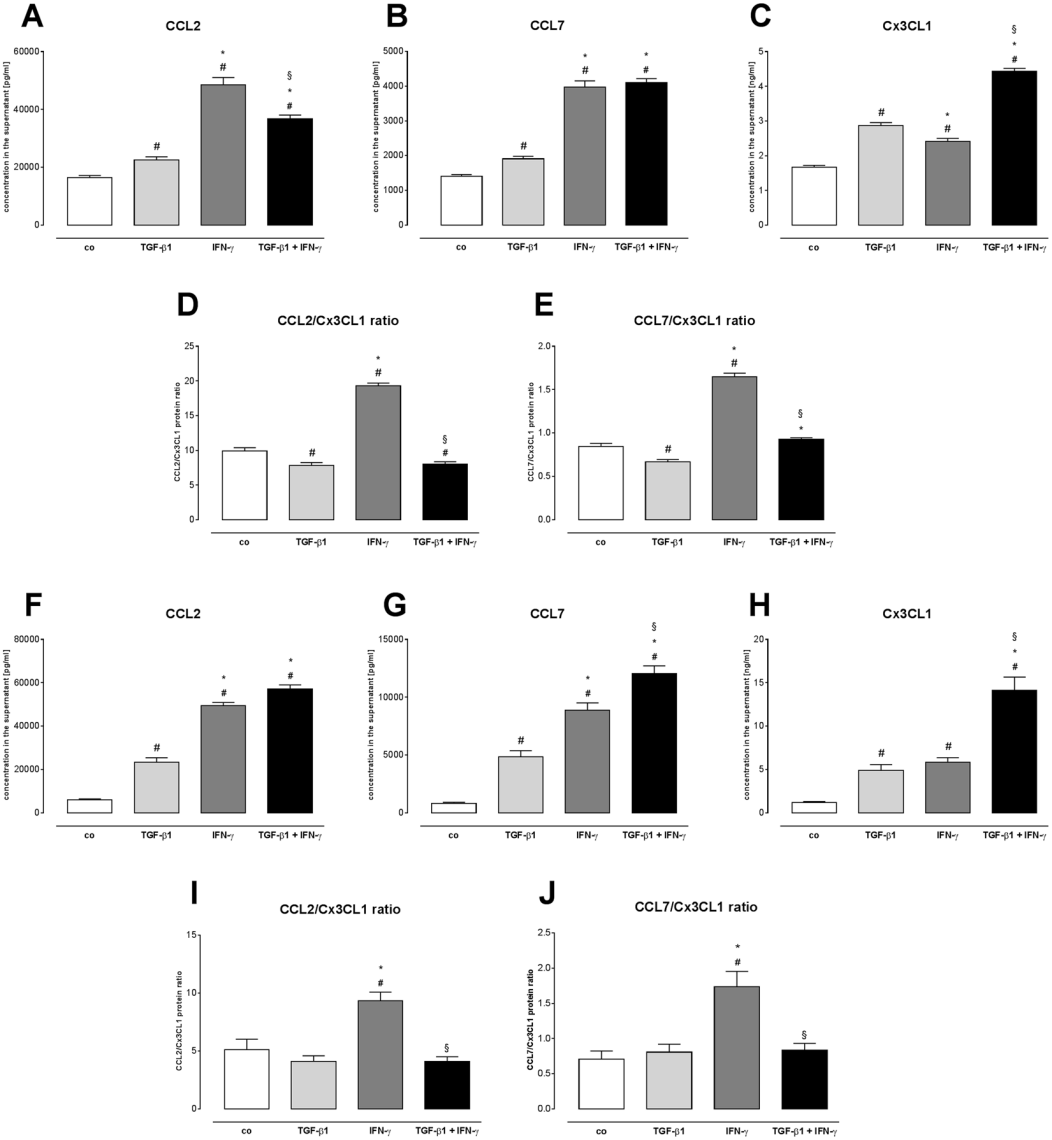
IFN-γ- and TGF-ß1-induced chemokine secretion and -modulated CCL2/Cx3CL1 and CCL2/Cx3CL1 protein ratio. (**A**–**C**) Protein levels of CCL2 (pg/ml), CCL7 (pg/ml), and Cx3CL1 (ng/ml) and (**D**,**E**) corresponding CCL2/Cx3CL1 and CCL2/Cx3CL ratio’s of control, TGF-ß1-, IFN-γ-, or TGF-β1 + IFN-γ-stimulated C4 fibroblasts after 72 h incubation time. In agreement, changes in secreted (**F**) CCL2 (pg/ml), (**G**) CCL7 (pg/ml) and (**H**) Cx3CL1 (ng/ml) and (**I,J**) CCL2/Cx3CL1 and CCL2/Cx3CL ratio’s from control, TGF-ß1-, IFN-γ-, or TGF-β1 + IFN-γ-stimulated cardiac fibroblasts were observed. Data are depicted as mean ± SEM from 3 independent experiments. Statistical analysis was performed by One way-ANOVA or Kruskal Wallis test (^#^p < 0.05 vs co, *p < 0.05 vs TGF-β1, ^§^p < 0.05 vs IFN-γ).

In view of getting *in vitro* insights in whether TGF-ß1 and IFN-γ influence the attraction of immune cells towards the heart during the remodelling process, chemokine production upon TGF-ß1, IFN-γ, and TGF-ß1 + IFN-γ stimulation of C4 fibroblasts and cardiac fibroblasts was examined (Fig. 3). In supernatants of C4 fibroblasts, 1.4-fold (p < 0.01), 1.4-fold (p < 0.01) and 1.7-fold (p < 0.0001) elevated CCL2, CCL7 and Cx3CL1 protein levels were observed after TGF-ß1 stimulation as compared respectively to control C4 fibroblasts (Fig. 3A–C). Interestingly, the addition of IFN-γ to TGF-ß1 further increased the secretion of CCL2, CCL7, and Cx3CL1 versus sole TGF-ß1 treatment (CCL2: 1.6-fold, p < 0.0001; CCL7: 2.2-fold, p < 0.0001; Cx3CL1: 1.5-fold, p < 0.0001). In accordance with the obtained results for C4 fibroblasts, cardiac fibroblasts also altered their chemokine release due to cytokine stimulation by 2.4-fold (p < 0.0001), 2.5-fold (p < 0.0001) and 2.9-fold (p < 0.0001) higher CCL2, CCL7, and Cx3CL1 protein levels upon TGF-ß1 + IFN-γ co-stimulation in comparison to sole TGF-ß1 treatment (Fig. 3F–H), respectively.

Since CCL2 and CCL7 are chemokines attracting pro-inflammatory monocytes and Cx3CL1 attracts anti-inflammatory monocytes^15^, we next calculated the ratio of CCL2 towards Cx3CL1 and CCL7 towards Cx3CL1 as indicative parameters of monocyte subset migration. In C4 fibroblasts, sole TGF-ß1 stimulation led to a 1.3-fold (p < 0.001) and 1.3-fold (p < 0.05) lower CCL2/Cx3CL1, and CCL7/Cx3CL1 ratio as compared respectively to the control fibroblasts (Fig. 3D,E), whereas addition of IFN-γ resulted in a 1.4-fold (p < 0.01) higher CCL7/Cx3CL1 ratio versus single TGF-ß1 treatment (Fig. 3D). No difference in CCL2/Cx3CL1 ratio was observed between supernatants of TGF-ß1 + IFN-γ and sole TGF-ß1-treated C4 fibroblasts (Fig. 3C). A similar regulation in CCL2/Cx3CL1 and CCL7/Cx3CL1 ratio was found for the supernatants of TGF-ß1- and/or IFN-γ-stimulated cardiac fibroblasts (Fig. 3I,J).

### IFN-γ reduces TGF-ß1-induced expression and activity of matrix metalloproteinase (MMP)-2

**Figure 4:**
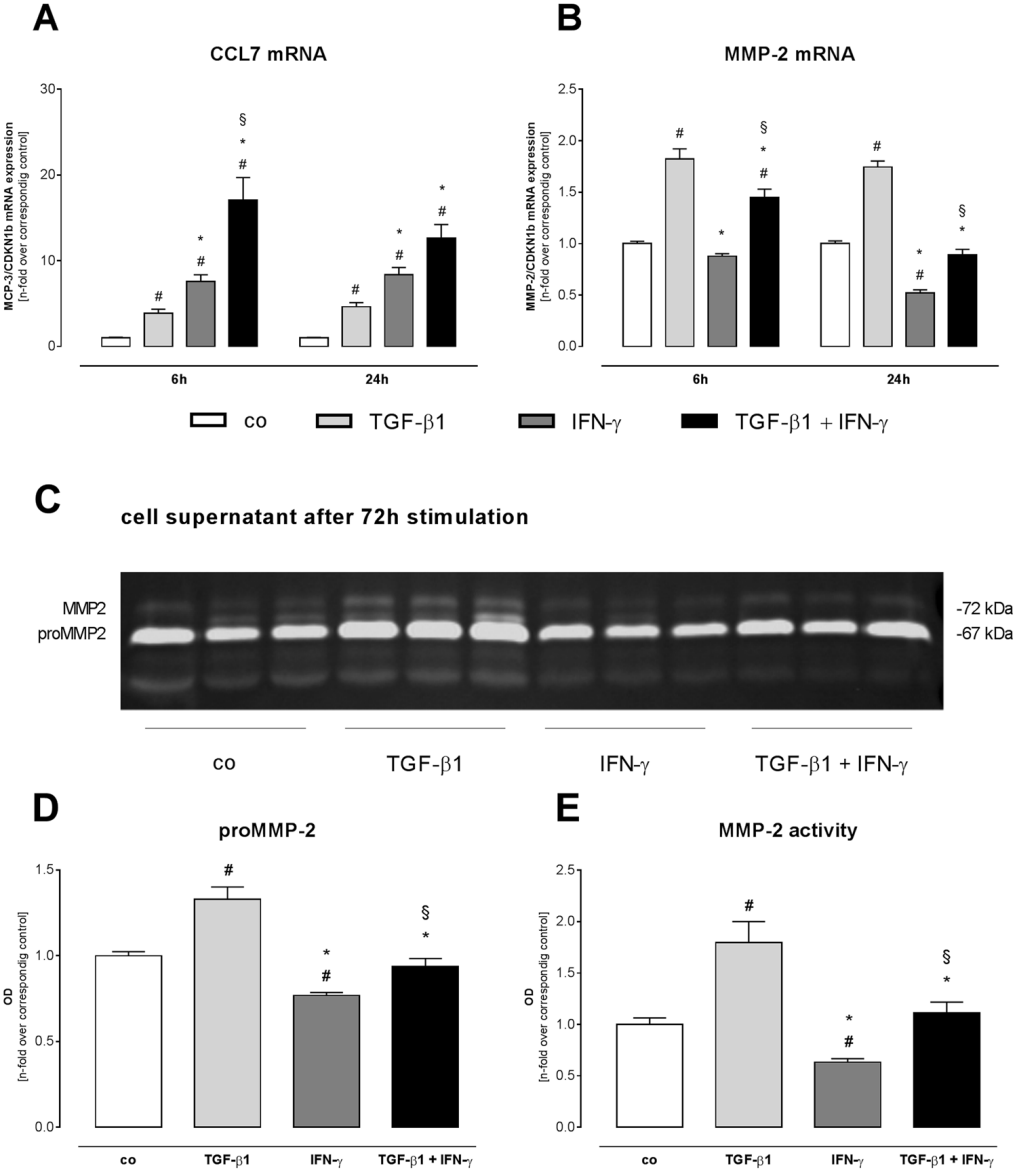
IFN-γ reduces TGF-ß1-induced expression and activity of MMP-2 in cardiac fibroblasts. Gene expression of CCL7 (**A**) and MMP-2 (**B**) after 6 and 24 h incubation time. To investigate the MMP-2 activity, zymography (**C**) of the cell culture supernatant was performed followed by quantification of proMMP-2 (**D**) and MMP-2 (**E**). Data are depicted as mean ± SEM from 3 independent experiments. Statistical analysis was performed by One way-ANOVA or Kruskal Wallis test (^#^p < 0.05 vs co, *p < 0.05 vs TGF-β1, ^§^p < 0.05 vs IFN-γ).

Given the relevance of MMP-2 on CCL7 cleavage^18^, we next analyzed the effects of TGF-ß1, IFN-γ, and TGF-ß1 + IFN-γ on the expression and activity of MMP-2 (Fig. 4). First, we evaluated their impact on CCL7 mRNA expression and confirmed the observed regulation of CCL7 on protein level. In detail, TGF-ß1 treatment for 24 h resulted in a 4.6-fold (p < 0.01) higher CCL7 mRNA level compared to control fibroblasts (Fig. 4A). The combined stimulation with TGF-ß1 + IFN-γ led to a 2.7-fold (p < 0.001) enhanced CCL7 expression level versus single TGF-ß1 administration.

In parallel, MMP-2 expression was 1.7-fold (p < 0.0001) induced after sole TGF-ß1 administration compared to control fibroblasts (Fig. 4B), whereas addition of IFN-γ to TGF-ß1 diminished MMP-2 mRNA levels by 2.0-fold (p < 0.0001). In line with the increased gene expression, proMMP-2 and MMP-2 activity were 1.3-fold (p < 0.01) and 1.8-fold (p < 0.01) higher after TGF-ß1 stimulation versus control fibroblasts (Fig. 4D,E), respectively. In comparison to TGF-ß1, proMMP-2 and MMP-2 activity was 1.4-fold (p < 0.0001) and 1.6-fold (p < 0.01) suppressed by the simultaneous stimulation with TGF-ß1 + IFN-γ, respectively.

### IFN-γ in combination with TGF-β1 induces migration of pro-inflammatory monocytes

**Figure 5:**
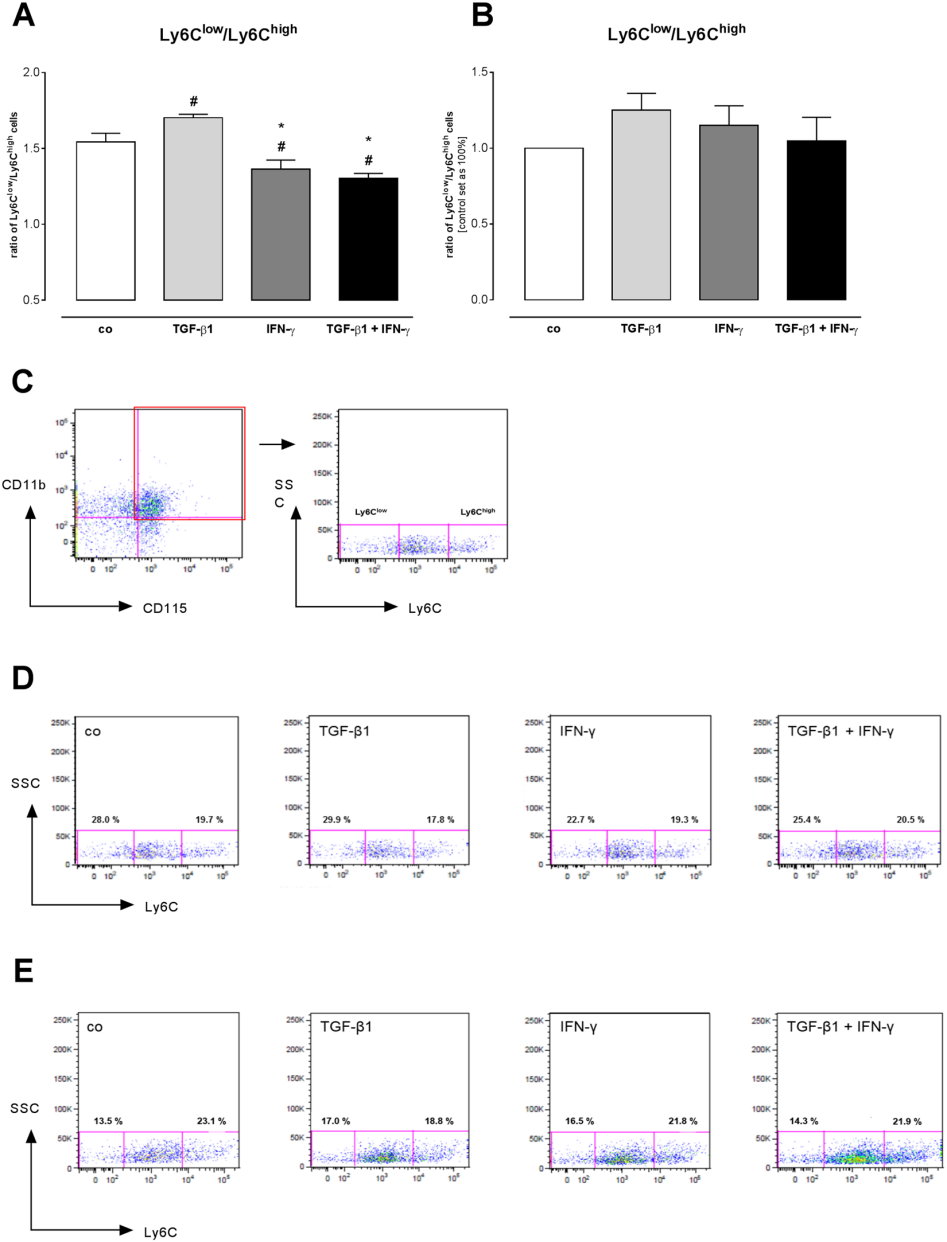
IFN-γ- and TGF-ß1-induced monocyte migration. (**A**) Flow cytometry analysis of CD11b^+^CD115^+^Ly6C^+^ stained cells shows differences between the ratios of Ly6C^low^/Ly6C^high^ monocytes versus the different conditioned media in C4 fibroblasts. (**B**) Indication for enhanced migration of Ly6C^low^ monocytes towards TGF-ß1-conditioned media of cardiac fibroblasts, whereas co-stimulation with TGF-β1 + IFN-γ increases the number of migrated Ly6C^high^ monocytes. (**C**) Gating strategy to define the amount of CD11b^+^CD115^+^Ly6C^+^ cells. (**D**) Representative dot blots for the migrated monocytes of the different conditioned media of stimulated C4 fibroblasts (n = 6/condition). (**E**) Representative dot blots for the migrated monocytes of the different conditioned media of stimulated cardiac fibroblasts (n = 3/condition). Data are shown as ratio of Ly6C^low^/Ly6C^high^ monocytes of 3 independent experiments. Statistical analysis was performed by One way-ANOVA or Kruskal Wallis test (^#^p < 0.05 vs co, *p < 0.05 vs TGF-β1, ^§^p < 0.05 vs IFN-γ).

To verify how the different CCL2, CCL7, and Cx3CL1 chemokine expression affects the attraction of different monocyte subsets, a migration assay was performed with splenocytes from control C57BL6/j mice towards the supernatants of TGF-ß1- and/or IFN-γ*-*stimulated C4 fibroblasts and cardiac fibroblasts (Fig. 5). The lower CCL2/Cx3CL1 and CCL7/Cx3CL1 ratio in the supernatant of TGF-ß1-stimulated versus control C4 fibroblasts was paralleled by a 1.1-fold (p < 0.05) higher ratio of Ly6C^low^ towards Ly6C^high^ migrated cells, indicating a lower number of attracted pro-inflammatory Ly6C^high^ monocytes compared to anti-inflammatory Ly6C^low^ monocytes (Fig. 5A). In contrast, the ratio of Ly6C^low^/Ly6C^high^ migrated monocytes was 1.3-fold (p < 0.0001) lower versus the supernatant of TGF-ß1 + IFN-γ- compared to TGF-ß1-stimulated fibroblasts. In agreement to the observed results for C4 fibroblasts, supernatant of TGF-β1-stimulated cardiac fibroblasts also attracted more Ly6C^low^ monocytes than Ly6C^high^ monocytes (Fig. 5B), whereas TGF-β1 + IFN-γ conditioned media mainly induced the migration of Ly6C^high^ monocytes.

## Discussion

The prominent findings of the present study are that TGF-ß1 induces the production of CCL2, CCL7, and Cx3CL1 in cardiac fibroblasts, which results in an enhanced migration of (anti-inflammatory) Ly6C^low^ monocytes. IFN-γ combined with TGF-ß1 induces a chemokine profile in cardiac fibroblasts in favor of attracting pro-inflammatory monocytes, as indicated by a reduction of the Ly6C^low^/Ly6C^high^ monocyte ratio.

Myocardial remodelling is a complex process, which involves multiple different steps^19^. After an initial damage to the heart, the immune system is activated and coordinates the function of several cell types, both of immune and non-immune origin, thereby affecting the pathological remodelling of the heart and the progression to heart failure^2^. In patients with chronic heart failure, serum levels of pro-inflammatory cytokines and chemokines are upregulated and correlate with the severity of the disease and the prognosis^20,21^. We previously demonstrated that IFN-γ decreases the differentiation of fibroblasts from the infarcted area in a mouse model of myocardial infarction, leading to decreased production of collagen I and III and impaired wound healing^11^. Based on this finding, we wanted to further explore the impact of IFN-γ on TGF-ß1-induced chemokine expression of cardiac fibroblasts and their subsequent potential of attracting monocyte subsets.

Cardiac fibroblasts get activated in the myocardium mainly by pro-fibrotic factors such as TGF-β1^22^ and differentiate into myofibroblasts, which are characterized by high expression of α-SMA, increased production of extracellular matrix proteins, mainly collagens^2^, and upregulated expression of the crosslinking enzymes Lox-1 and LoxL-2, which on their turn promote fibroblast-to-myofibroblast activation^23–25^. In accordance with previous reports showing anti-fibrotic effects of IFN-γ^26–28^, we demonstrate that simultaneous stimulation of cardiac fibroblasts with TGF-β1 and IFN-γ resulted in lower gene and protein expression of collagens and α-SMA, and led to lower expression levels of Lox-1 as well as LoxL-2. Interestingly, Lox-1 is besides its role in crosslinking also known to resolve inflammation by reducing CCL2 expression^29^. In accordance to this negative correlation between Lox-1 and CCL2, higher CCL2 levels were observed in the conditioned media of TGF-ß1 + IFN-γ- versus sole TGF-ß1-stimulated cardiac fibroblasts. However, Lox-1 also has own chemoattractant potential^30^. This hints to the complex role of Lox in inflammation and monocyte attraction on the one hand, and further confirms cardiac fibroblasts as inflammatory supporter cells on the other hand^2,3^. Further investigations are needed to clarify the specific role of cardiac fibroblast-derived Lox-1 and/or LoxL-2 in the attraction of monocyte (subsets).

Besides CCL2, also CCL7 and Cx3CL1 levels were enriched in cell culture supernatant of TGF-ß1-stimulated cardiac fibroblasts compared to control fibroblasts. Since MMP-2 is known to degrade CCL7^31^, we evaluated whether the found CCL7 protein expression could be explained by changes in MMP-2 activity. Conforming Westermann *et al*.^22^, who demonstrated an increase in MMP-2 activity upon TGF-ß1 stimulation of cardiac fibroblasts derived from endomyocardial biopsies of HFpEF patients, TGF-ß1 increased MMP-2 expression and activity. In contrast to that, co-stimulation of TGF-ß1 with IFN-γ reduced MMP-2 expression and activity, which might explain the higher CCL7 expression under these stimuli versus TGF-ß1 stimulation. This finding is in line with the observation that reduced MMP-2 activity in viral-induced myocarditis was associated with increased levels of CCL7/MCP-3 and translated in exacerbation of cardiac immune cell infiltration^32^. In addition, we demonstrated that TGF-ß1 + IFN-γ further promotes an inflammatory phenotype of cardiac fibroblasts, as indicated by higher expression of the cell adhesion molecules ICAM-1 and VCAM-1, and of the inflammatory cytokine TNF-α compared to sole TGF-ß1 supplementation.

Regarding cell migration, TGF-ß1-stimulated cardiac fibroblasts exhibited lower CCL2/Cx3CL1 and CCL7/Cx3CL1 ratios in their conditioned media compared to control fibroblasts. In accordance to the chemoattractant potential of CCL2 and CCL7 attracting pro-inflammatory Ly6C^high^ monocytes, whereas Cx3CL1 mainly attracts anti-inflammatory Ly6C^low^ monocytes^15,33^, this TGF-ß1-induced chemokine profile was reflected in the attraction of a higher number of anti-inflammatory Ly6C^low^ cells compared to pro-inflammatory Ly6C^high^ monocytes. Simultaneous stimulation with TGF-ß1 and IFN-γ promoted the pro-inflammatory phenotype, as shown by the higher ratio of CCL7/Cx3CL1 compared to sole TGF-ß1 treatment. This was reflected in the enhanced migration of pro-inflammatory Ly6C^high^ monocytes towards this media. Similar results were observed for rat cardiac fibroblasts by which TGF-β1 conditioned media induced M2 macrophage polarization, whereas LPS-treated media favored M1 macrophage polarization^34^. These findings are further conforming own data from previous experimental CVB3-induced myocarditis mice, which exhibited elevated CCL2/Cx3CL1 and CCL7/Cx3CL1 ratios, and were associated with an increased cardiac presence of pro-inflammatory towards anti-inflammatory monocytes^13^. Finally, the TGF-ß1- and IFN-γ-induced chemokine profile of cardiac fibroblasts and subsequent attraction of pro- and anti-inflammatory monocytes corresponds to the sequential attraction of Ly6C^high^ and Ly6C^low^ monocytes post myocardial infarction^15^ and their function in cardiac wound healing and scar formation^33,35^.

**Figure 6:**
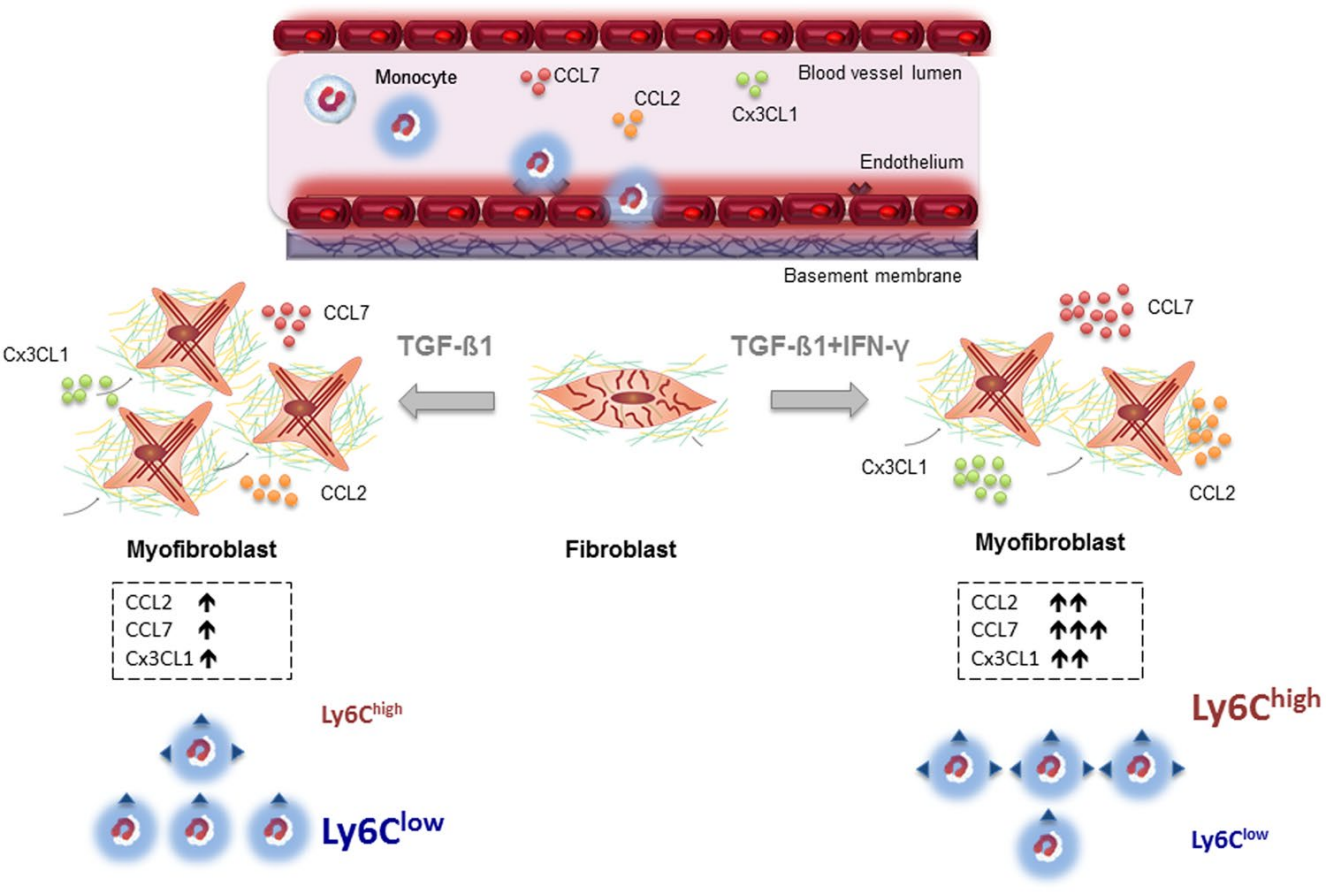
Hypothetical scheme representing how TGF-ß1 and IFN-γ affect the chemokine expression and subsequent monocyte migration of cardiac fibroblasts. The Figure illustrates the transdifferentiation process of fibroblasts towards myofibroblasts under TGF-ß1 and TGF-ß1 + IFN-γ conditions. Depending on the stimulus, the specific chemokine profile (CCL2, CCL7, and Cx3CL1) of the myofibroblasts results in a more pronounced attraction of Ly6C^low^ monocytes (TGF-ß1 stimulation) or of Ly6C^high^ monocytes (TGF-ß1 + IFN-γ stimulation). The Figure uses elements previously published by Van Linthout *et al*.^2^  Stands for Ly6C.

In conclusion, TGF-ß1 boosts chemokine expression stimulating the attraction of Ly6C^low^ monocytes, whereas IFN-γ in conjunction with TGF-ß1, particularly attracts Ly6C^high^ monocytes (Fig. 6). It is tempting to speculate that primed fibroblasts contribute to the repair process via attracting specific monocytes subsets, and that this homing is regulated via a balance between pro-inflammatory (IFN-γ) versus pro-fibrotic (TGF-ß1) factors. It is clear that during the pathogenesis of heart failure more cytokines and growth factors are involved in the cardiac remodelling process than the pro-inflammatory IFN-γ and the pro-fibrotic TGF-ß1. Nevertheless, this study is the first study to our knowledge, which points out the interplay among IFN-γ and TGF-ß1 on cardiac fibroblasts influencing the functional phenotype of the (myo)fibroblasts with respect to the attraction of monocyte subsets. However, further studies are warranted to get further insights into the complex regulation of fibroblasts in the inflammatory and remodelling process of distinct cardiac disorders.

## Materials and Methods

### Isolation and expansion of cardiac fibroblasts

Murine cardiac fibroblasts were obtained from the LV of 8–12 weeks old healthy male C57BL6/j mice (n = 6, Charles River, Sulzfeld, Germany), cut in approximately 2 mm large pieces and fixed in 12-well culture plates (n = 1/well). Therefore, mice were euthanized via cervical dislocation, which is approved by the local ethics committee (Landesamt für Gesundheit und Soziales, Berlin, T0098/11) and in accordance to the European legislation for the Care and Use of Laboratory Animals (Directive 2010/63/EU). The outgrowing cardiac fibroblasts were cultured in Dulbecco’s modified eagle medium (DMEM; PAA, Cölbe; Germany) containing 20% fetal calf serum (FCS; Biochrom, Berlin, Germany), 100 U/mL penicillin and 100 µg/mL streptomycin (both PAA, Cölbe, Germany) at 37 °C with 95% air and 5% CO_2_. Stimulation experiments were performed with cells in passage 4 to 8.

### Culture and stimulation of C4 fibroblasts

Murine C4 fibroblasts, a murine fibroblast cell line derived from embryonic BALB/c mice by SV40 infection *in vitro*^36^, were cultured in Iscove medium (Sigma Aldrich, Munich, Germany) supplemented with 10% FCS (Biochrom) and 1% penicillin/streptomycin (Biochrom). For stimulation experiments, 60,000 cells/well were plated in 12-well-plates. One day after plating, cells were washed once with phosphate buffered saline (PBS; Thermo Fisher Scientific, Waltham, Massachusetts, USA), and starvation medium (Iscove medium + 0.05% FBS + 1% P/S) was added. After 24 hours (h), cells were stimulated with or without 5 ng/mL TGF-β1 (Peprotech, Hamburg, Germany), 10 ng/mL IFN-γ (Peprotech) or both in starvation medium. 72 h after stimulation, the supernatant was harvested, centrifuged (10 minutes (min) at 4 °C, 12000 × g) and aliquoted to further investigate chemokine secretion and perform chemotaxis assays.

### Stimulation experiments of cardiac fibroblasts

For stimulation experiments, murine cardiac fibroblasts were seeded out in 24-well culture plates (30,000 cells/well) for RNA isolation and immunofluorescence or 12-well culture plates (10,000 cells/well) for protein analysis and zymography. Stimulation with TGF-β1 (Peprotech) was chosen to induce differentiation of cardiac fibroblasts into myofibroblasts, mimicking the environment present during cardiac remodelling^37^. When reaching 80% confluence, cells were serum starved in DMEM (PAA) containing 0.5% FCS (Biochrom), 100 U/mL penicillin and 100 µg/mL streptomycin (both PAA), overnight. After washing once with PBS (PAA), cells were incubated with IFN-γ (Peprotech) at a final concentration of 10 ng/mL or with 5 ng/mL TGF-β1 or both up to 72 h. Unstimulated control cells were incubated for the same time period with serum-starved DMEM (PAA). Stimulation experiments were carried out with n = 6 wells per stimulation condition and repeated three times.

### RNA Isolation, cDNA transcription and real-time-PCR

After 6 and 24 h incubation, cells were washed once with PBS (PAA). RLT-Buffer containing 1% β-mercaptoethanol was added and cell lysates were stored at −80 °C until further investigations. After thawing, 70% ethanol was added and RNA was isolated using the RNeasy kit (Qiagen, Hilden, Germany). Next, 250 ng of total RNA was reverse transcribed to cDNA with the high-capacity kit (Applied Biosystems, Darmstadt, Germany). Gene expression analysis was performed within the ViiA7 TaqMan System (Life technologies, Darmstadt, Germany) by using 5 μL of the gene expression master mix (Applied Biosystems), 0.5 μL of the gene expression assay (Applied Biosystems) and 1 μL of the cDNA in a final volume of 10 μL. Following gene expression assays were used: Col1a1 (Mm01302043_g1), Col3a1 (Mm00802331_m1), α-SMA (Mm00725412_s1), TGF-β1 (Mm00441724_m1). Lox-1 (Mm00495386_m1), LoxL-2 (Mm00804740_m1), MMP-2 (Mm00439505_m1), CCL7 (Mm00443113_m1), ICAM-1 (Mm01320970_m1), VCAM-1 (Mm00516023_m1), TNF-α (Mm00443258_m1) and IL-6 (Mm00446190_m1). For the quantification of the relative mRNA expression, all data were normalized to CDKN1b (Mm00438167_g1) as an internal control expressed in the 2^−ΔCt^ formula. For comparison of the different response to TGF-β1 and IFN-γ treatment, mRNA data are expressed as 2^−ΔΔCt^ by normalization to the corresponding untreated control.

### Immunofluorescence staining

Murine cardiac fibroblasts were incubated with external stimuli for up to 72 h. After washing once with PBS (PAA), cells were fixed with 4% paraformaldehyde for 10 min. Cells were permeabilized with Triton X-100 and incubated with avidin blocking solution for 30 min. Then, cells were treated with the primary antibody α-SMA (1:50; Dako, Hamburg, Germany) in the presence of biotin solution for 90 min. Afterwards, fibroblasts were washed twice followed by 1 h incubation with the biotinylated secondary antibody (1:250; Dianova, Hamburg, Germany). As described previously^11^, cells were finally incubated 30 min with Cy3-conjugated streptavidin (1:250; Jackson ImmunoResearch Laboratories Inc., West Grove, PA, USA) for fluorescence visualization and with Diamidinphenylindol (DAPI; 1:100; Invitrogen, Darmstadt, Germany) as nuclear staining. All incubations were performed at room temperature.

### Protein extraction and western blot analysis

After 72 h incubation time, supernatants were collected and cells were washed with PBS. Then, fibroblasts were lysed with 150 µL cell lysis buffer containing a protease and phosphatase inhibitor cocktail (both Sigma-Aldrich). Denaturized proteins were electrophoresed on 10% SDS-polyacrylamide gels, followed by transfer onto PVDF membranes and blocking with 5% skim milk for 30 min at room temperature. Blots were incubated over night with primary antibodies against collagen I (1:1000; Merck Millipore, Darmstadt, Germany) or GAPDH (1:1000; Cell Signaling, Frankfurt am Main, Germany) at 4 °C. The bound antibodies were detected by the respective horseradish peroxidase-conjugated secondary antibodies (1:2500, Cell signaling), which were visualized with super signal solution (Thermo Scientific, Darmstadt, Germany). The resulted chemiluminescence was measured by using the Chemo Star Imager camera system (INTAS Science Imaging Instruments, Göttingen, Germany). The relevant blots were analyzed by ImageJ software (NIH, Maryland, USA). For quantification of the protein levels, GAPDH was used as an internal control for each sample.

### Zymography

Gelatin zymography from cell culture supernatant of stimulated cardiac fibroblasts was performed to determine the gelatinolytic activities of MMP-2 in response to TGF-β1 and IFN-γ. After 72 h incubation, supernatants were collected and mixed with 5× sample buffer. Samples were loaded on 10% SDS-polyacrylamide gels copolymerized with 1 mg/mL of gelatine (VWR, Darmstadt, Germany). Following electrophoresis, gels were washed in renaturing buffer with gentle shaking for 20 min. Digestion of gelatine was carried out twice in developing buffer, at first for 20 min at room temperature and then for 18 h at 37 °C. Next, the solution was replaced and gels were incubated again with cold developing buffer for 20 min. After development, gels were stained with 0.5% coomassie G250 blue solution (Sigma-Aldrich) and finally slowly destained with destain solution. Recording of images was carried out under UV light using the BioDoc 2.0 Imaging system (UVP, Cambridge, UK). ImageJ (NIH) was used for quantification of the bands and expressed as n-fold to the corresponding control.

### ELISA

To determine the secreted amount of CCL2 and CCL7 in the cell culture media of stimulated cells, the respective ELISA kits (both Peprotech) were used. In brief, ELISA microplates were coated overnight with the capture antibody (1:100) followed by 4 times washing with PBS + 0.05% Tween 20, and blocking with the blocking buffer for at least 1 h. Next, standards or samples were added to the wells for 2 h, followed by washing and incubation with the detection antibody (1:200) for additional 2 h. After washing, the avidin-HRP conjugate (1:2000) was added to each well. For color development, ABTS liquid substrate (Sigma-Aldrich) was used and the absorbance was measured at 405 nm. To determine the secreted amount of Cx3CL1, the murine Cx3CL1 ELISA kit (Abcam, Cambrigde, UK) was used. The absorbance was measured at the announced wavelength according to the manufacturer’s protocol.

### CytoSelect™ cell migration assay

To determine the chemotactic potential of the supernatants of the (un)stimulated fibroblasts, a cell migration assay (Cell Biolabs, San Diego, USA) was performed according to the manufacturer’s protocol. Therefore, splenocytes of 8–12 weeks old healthy male C57BL6/j mice (n = 10, Charles River) were isolated, pooled and placed at a density of 0.1 × 10^6^ cells/ml on the upper membrane chamber. Under sterile conditions, 150 µL of the respective supernatants (=further named as conditioned media) were added to the wells of the feeder tray to explore chemotaxis. After 24 h incubation time, migratory cells were harvested and further analyzed by flow cytometry. For migration experiments versus conditioned media of C4 fibroblasts, migrated splenocytes of 8 wells/condition were pooled to obtain enough cells for subsequent flow cytometry. The migration assay was repeated 6 times/condition (n = 6). With respect to cardiac fibroblasts, migrated splenocytes of 4 wells/condition were pooled and performed three times (n = 3).

### Flow cytometry

To investigate the different monocyte subsets within the migrated splenocytes, flow cytometry was performed. In general, cells were first washed with PBS and after centrifugation stained at 4 °C and 30 min for CD11b (2:5), CD115 (1:50) and Ly6C (1:20) to determine the fraction of migrated pro-inflammatory (CD11b^+^CD115^+^Ly6C^high^) and patrolling/reparative (CD11b^+^CD115^+^Ly6C^low^) monocytes. Antibodies against CD11b, CD115 and Ly6C were purchased from Biolegend (London, UK). Sample analysis was performed using the MACSQuant Analyzer (Miltenyi Biotec, Bergisch Gladbach, Germany) and flow cytometry data were analyzed with FlowJo 8.7. software (FlowJo, LLC, RO, USA).

### Statistical analysis

Data are shown as mean ± SEM and all statistical analyses were performed using GraphPad Prism 7.0 software (GraphPad Software Inc, La Jolla, USA). First, data were tested with Shapiro-Wilk test for normal distribution. For the comparison of the different stimuli, parametric data were tested with ordinary One-way-ANOVA (Fisher’s LSD post hoc test) or with Kruskal-Wallis test (uncorrected Dunn’s post hoc test) in case of non-parametric data sets. Statistical differences were assessed significant at p < 0.05.

## References

[1] Weber KT, Sun Y, Bhattacharya SK, Ahokas RA, Gerling IC (2013). Myofibroblast-mediated mechanisms of pathological remodelling of the heart. Nat Rev Cardiol.

[2] Van Linthout S, Miteva K, Tschope C (2014). Crosstalk between fibroblasts and inflammatory cells. Cardiovasc Res.

[3] Lindner D (2014). Cardiac fibroblasts support cardiac inflammation in heart failure. Basic Res Cardiol.

[4] Frangogiannis NG (2012). Regulation of the inflammatory response in cardiac repair. Circ Res.

[5] Chen W, Frangogiannis NG (2013). Fibroblasts in post-infarction inflammation and cardiac repair. Biochim Biophys Acta.

[6] van Nieuwenhoven FA, Turner NA (2013). The role of cardiac fibroblasts in the transition from inflammation to fibrosis following myocardial infarction. Vascul Pharmacol.

[7] Spillmann F, Miteva K, Pieske B, Tschope C, Van Linthout S (2015). High-Density Lipoproteins Reduce Endothelial-to-Mesenchymal Transition. Arterioscler Thromb Vasc Biol.

[8] Akhurst RJ, Hata A (2012). Targeting the TGFbeta signalling pathway in disease. Nat Rev Drug Discov.

[9] van den Borne SW (2010). Myocardial remodeling after infarction: the role of myofibroblasts. Nat Rev Cardiol.

[10] Zhu J, Paul WE (2008). CD4 T cells: fates, functions, and faults. Blood.

[11] Savvatis K (2014). Interleukin-23 deficiency leads to impaired wound healing and adverse prognosis after myocardial infarction. Circ Heart Fail.

[12] Frangogiannis NG (2014). The inflammatory response in myocardial injury, repair, and remodelling. Nat Rev Cardiol.

[13] Miteva K (2017). Mesenchymal Stromal Cells Modulate Monocytes Trafficking in Coxsackievirus B3-Induced Myocarditis. Stem Cells Transl Med.

[14] Nahrendorf M, Pittet MJ, Swirski FK (2010). Monocytes: protagonists of infarct inflammation and repair after myocardial infarction. Circulation.

[15] Nahrendorf M (2007). The healing myocardium sequentially mobilizes two monocyte subsets with divergent and complementary functions. J Exp Med.

[16] Muller I (2017). CX3CR1 knockout aggravates Coxsackievirus B3-induced myocarditis. PLoS One.

[17] Sobral LM, Montan PF, Martelli-Junior H, Graner E, Coletta RD (2007). Opposite effects of TGF-beta1 and IFN-gamma on transdifferentiation of myofibroblast in human gingival cell cultures. J Clin Periodontol.

[18] Lafleur MA, Handsley MM, Edwards DR (2003). Metalloproteinases and their inhibitors in angiogenesis. Expert Rev Mol Med.

[19] Opie LH, Commerford PJ, Gersh BJ, Pfeffer MA (2006). Controversies in ventricular remodelling. Lancet.

[20] Aukrust P (1999). Cytokine network in congestive heart failure secondary to ischemic or idiopathic dilated cardiomyopathy. Am J Cardiol.

[21] Torre-Amione G (1996). Proinflammatory cytokine levels in patients with depressed left ventricular ejection fraction: a report from the Studies of Left Ventricular Dysfunction (SOLVD). J Am Coll Cardiol.

[22] Westermann D (2011). Cardiac inflammation contributes to changes in the extracellular matrix in patients with heart failure and normal ejection fraction. Circ Heart Fail.

[23] Counts DF, Evans JN, Dipetrillo TA, Sterling KM, Kelley J (1981). Collagen lysyl oxidase activity in the lung increases during bleomycin-induced lung fibrosis. J Pharmacol Exp Ther.

[24] Yang J (2016). Targeting LOXL2 for cardiac interstitial fibrosis and heart failure treatment. Nat Commun.

[25] Gonzalez-Santamaria J (2016). Matrix cross-linking lysyl oxidases are induced in response to myocardial infarction and promote cardiac dysfunction. Cardiovasc Res.

[26] Jimenez SA, Freundlich B, Rosenbloom J (1984). Selective inhibition of human diploid fibroblast collagen synthesis by interferons. J Clin Invest.

[27] Gurujeyalakshmi G, Giri SN (1995). Molecular mechanisms of antifibrotic effect of interferon gamma in bleomycin-mouse model of lung fibrosis: downregulation of TGF-beta and procollagen I and III gene expression. Exp Lung Res.

[28] Song YL, Ford JW, Gordon D, Shanley CJ (2000). Regulation of lysyl oxidase by interferon-gamma in rat aortic smooth muscle cells. Arterioscler Thromb Vasc Biol.

[29] Onoda M (2010). Lysyl oxidase resolves inflammation by reducing monocyte chemoattractant protein-1 in abdominal aortic aneurysm. Atherosclerosis.

[30] Lazarus HM, Cruikshank WW, Narasimhan N, Kagan HM, Center DM (1995). Induction of human monocyte motility by lysyl oxidase. Matrix Biol.

[31] McQuibban GA (2000). Inflammation dampened by gelatinase A cleavage of monocyte chemoattractant protein-3. Science.

[32] Westermann D (2011). Reduced degradation of the chemokine MCP-3 by matrix metalloproteinase-2 exacerbates myocardial inflammation in experimental viral cardiomyopathy. Circulation.

[33] Stievano L, Piovan E, Amadori A (2004). C and CX3C chemokines: cell sources and physiopathological implications. Crit Rev Immunol.

[34] Humeres, C. *et al*. Cardiac fibroblast cytokine profiles induced by proinflammatory or profibrotic stimuli promote monocyte recruitment and modulate macrophage M1/M2 balance *in vitro*. *J Mol Cell Cardiol*, 10.1016/j.yjmcc.2016.10.014 (2016). 10.1016/j.yjmcc.2016.10.014 27983968

[35] Dewald O (2005). CCL2/Monocyte Chemoattractant Protein-1 regulates inflammatory responses critical to healing myocardial infarcts. Circ Res.

[36] Schwarz K (2000). The selective proteasome inhibitors lactacystin and epoxomicin can be used to either up- or down-regulate antigen presentation at nontoxic doses. Journal of immunology.

[37] Desmouliere A, Geinoz A, Gabbiani F, Gabbiani G (1993). Transforming growth factor-beta 1 induces alpha-smooth muscle actin expression in granulation tissue myofibroblasts and in quiescent and growing cultured fibroblasts. J Cell Biol.

